# Hyperventilation in Adult TBI Patients: How to Approach It?

**DOI:** 10.3389/fneur.2020.580859

**Published:** 2021-01-28

**Authors:** Elisa Gouvea Bogossian, Lorenzo Peluso, Jacques Creteur, Fabio Silvio Taccone

**Affiliations:** Intensive Care Department, Erasmus Hospital, Université Libre de Bruxelles, Brussels, Belgium

**Keywords:** traumatic brain injury, hyperventilation, hypocapnia, intracranial hypertension, cerebral ischemia

## Abstract

Hyperventilation is a commonly used therapy to treat intracranial hypertension (ICTH) in traumatic brain injury patients (TBI). Hyperventilation promotes hypocapnia, which causes vasoconstriction in the cerebral arterioles and thus reduces cerebral blood flow and, to a lesser extent, cerebral blood volume effectively, decreasing temporarily intracranial pressure. However, hyperventilation can have serious systemic and cerebral deleterious effects, such as ventilator-induced lung injury or cerebral ischemia. The routine use of this therapy is therefore not recommended. Conversely, in specific conditions, such as refractory ICHT and imminent brain herniation, it can be an effective life-saving rescue therapy. The aim of this review is to describe the impact of hyperventilation on extra-cerebral organs and cerebral hemodynamics or metabolism, as well as to discuss the side effects and how to implement it to manage TBI patients.

## Introduction

Intracranial hypertension (ICHT) is the most critical and potentially devastating complication in traumatic brain injury (TBI) patients (1). Since the skull is a rigid compartment, the total volume of the intracranial contents, i.e., brain tissue, blood, and cerebral spinal fluid, will remain constant over time. An increase in the volume of one of these components is initially compensated by shifting parts of the others (i.e., compression of the cerebral venous system can decrease global cerebral blood volume; increased CSF reabsorption and CSF displacement toward the basal cisterns and spinal compartment can decrease the CSF volume); when these mechanisms can no longer compensate for further volume changes intracranial pressure (ICP) will rapidly rise (2). Both the duration of ICHT and the absolute maximum value of ICP have an impact on patients' outcome (3); therefore, therapies aimed at controlling ICP and minimizing the ICHT burden are the cornerstone of TBI management (4).

Although different therapeutic interventions are available, none of them has shown a significant impact on patients' outcome and some potential side effects may limit their use. Modulation of arterial carbon dioxide pressure (PaCO_2_) has been used since decades in neuro-anesthesia and in neuro-intensive care, because lowering PaCO_2_ (i.e., hypocapnia) through increased minute volume ventilation (i.e., hyperventilation) can rapidly contribute to reduce the volume of the swollen brain and help control ICP (5); these effects are mediated by cerebral vasoconstriction and reduction in cerebral blood flow (CBF) and cerebral blood volume (CBV) (1, 2).

Although commonly used, hyperventilation has not been extensively supported by robust evidence, its effects might be transient and may not improve the probability of neurological recovery (3). Moreover, by decreasing CBF, hyperventilation may trigger or enhance brain ischemia (4, 5). In addition, hyperventilation has some extra-cerebral effects that may negatively impact patients' outcome (6). As such, if hyperventilation is frequently employed in TBI patients (7), the potential risks associated with this therapy require an optimal understanding on how to manage PaCO_2_ in TBI patients.

The aim of this review is to describe the effects of hyperventilation on brain physiology and to discuss its use in the management of TBI patients. Only studies focusing on controlled hyperventilation (i.e., modification of minute ventilation in TBI patients treated with mechanical ventilation and controlled modes) have been evaluated, while pre-hospital hyperventilation or spontaneous hyperventilation will not be discussed.

## Hyperventilation, Hypocapnia and Systemic Effects

Hyperventilation is characterized by elevated minute alveolar ventilation, which can be secondary to an increase of tidal volume and/or respiratory rate, if the dead space remains constant. This condition is typically observed as a physiological response to hypoxemia, systemic inflammation, chest trauma, or pain; however, in the setting of TBI management, “controlled hyperventilation” is a modification of minute ventilation to obtain hypocapnia (i.e., PaCO_2_ <38 mmHg) in order to manipulate cerebral hemodynamics and compliance (8). In this setting, acceptable ranges of PaCO_2_ in clinical practice are considered between 35 and 45 mmHg at sea level (8); when hyperventilation is applied, it can classified into moderate (PaCO_2_ 31–35 mmHg), forced (PaCO_2_ 26–30 mmHg), or intensified forced (PaCO_2_ <26 mmHg), according to PaCO_2_ levels (9).

There are several non-cerebral effects related to this therapeutic strategy (Table 1); as TBI patients often have lung injury (7, 10, 11) due to micro-aspiration, pneumonia or lung contusions (12), promoting hyperventilation by increasing tidal volume can induce ventilator-induced lung injury (VILI) (13) and potentially delay pulmonary healing or worsen outcome (14–16). Moreover, hyperventilation may increase intra-thoracic pressure, which would favor right ventricular dysfunction or, in hypovolemic patients, cause an impairment of the venous return and decrease cardiac output (17). Moreover, hypocapnia compromises coronary blood flow and is associated with an increased risk of myocardial ischemia (18) and the development of arrhythmias (19). Prolonged hyperventilation is associated with respiratory alkalosis (20); alkalemia would shift the oxygen dissociation curve of hemoglobin toward the left, increasing the hemoglobin affinity for oxygen and compromising tissue oxygen delivery (18). Hypocapnia and respiratory alkalosis also lead to pulmonary vasodilation (19) and bronchoconstriction (21), which result in ventilation to perfusion (V/Q) mismatch and secondary hypoxemia in TBI patients with pre-existing lung injury. In animal studies, hypocapnia also decreased surfactant production (22) and increases the permeability of the alveolo-capillary barrier (23), although this has not well-demonstrated in humans. Hyperventilation may also increase intra-abdominal pressure, which can secondarily increase ICP (24); hypocapnia decreases blood flow to the kidneys, skin and muscles tissues and increases platelet adhesion and aggregation (12). Finally, hyperventilation is often associated with electrolytes disturbances, such as hypokalemia, hypocalcemia, and hypophosphatemia (12). Taken all together, these findings suggest that controlled hyperventilation and hypocapnia should be applied with extreme caution to all critically ill patients, because of several negative effects on targets organs.

**Table 1:** Potential side effects associated with hyperventilation in the human setting.

**Systemic**	**Cerebral**
Ventilation-induced lung injury	Cerebral vasoconstriction
Right ventricular dysfunction	Reduced CBF
Reduced cardiac output	Reduced CBV
Myocardial ischemia	Brain hypoxia
Cardiac arrhythmias	Increased neuronal excitability
Tissue hypoxia	Reduced epileptic threshold
Lung V/Q mismatch	Increased release of excitatory amino-acids
Increased intrabdominal pressure	Increased dopamine levels
Reduced renal flow	Altered membrane cell synthesis
Reduced skin flow	
Reduced muscular flow	
Increased platelet adhesion	
Increased platelet aggregation	
Hypokalemia, hypocalcemia, and hypophosphatemia	

*V/Q, ventilation to perfusion ratio; CBF, cerebral blood flow; CBV, cerebral blood volume*.

## Effects of Hyperventilation and Hypocapnia on Brain Physiology and Metabolism

The brain has a high energy requirement, being responsible for 20% of total body oxygen consumption (25). Since the brain is incapable of storing energy, rapid adjustments of CBF are essential to maintain an adequate supply of oxygen and nutrients to brain tissue (26). Several mechanisms, collectively called “cerebral autoregulation,” are effective to keep CBF within the necessary values to meet the cerebral energetical demand (26). As such, CBF is heterogenous and varies according to the metabolic activity of each cerebral region (27); resistance arterioles contract and dilate to regulate CBF in response to different stimuli, such as blood pressure, blood viscosity, transmural pressure, metabolic demand, tissue pH, and electrolytes or PaCO_2_ (28).

In particular, these resistance arterioles respond to variations in PaCO_2_ between 20 and 60 mmHg by contracting (i.e., hypocapnia) or dilating (i.e., hypercapnia) (2), a phenomenon called “cerebro-vascular CO_2_ reactivity.” This response to PaCO_2_ variations is probably pH-mediated (18) (i.e., low pH or high H^+^ concentrations will promote vasodilation, while high pH and low H^+^ vasoconstriction), and is proportionally more relevant with hypercapnia than with hypocapnia (29). Around 70% of the CBV is located within the venous system and is not affected by changes in PaCO_2_; therefore, changes in CBV following hyperventilation are restricted to the arterial component and are associated with a decrease in CBF (18). In particular, for each mmHg-decrease in PaCO_2_, there is an approximate decrease of 3% in CBF (2), although the impact of hypocapnia on ICP is less pronounced (5).

Hyperventilation can also result in brain hypoxia (Table 1). The main mechanism suggested is the reduction of oxygen supply due global and/or regional hypoperfusion caused by the reduction in CBF (30, 31). Moreover, due to the above-mentioned systemic effects, hypocapnia can lead to additional VILI, with impaired gas exchanges and hypoxemia, and can alter the oxygen hemoglobin dissociation curve, with reduced oxygen delivery (18).

Finally, alkalemia and hypocapnia increase neuronal excitability (32), reduce the epileptic threshold and/or prolong convulsive activities (33). In animal studies, hypocapnia led to an increased cerebral consumption and depletion of local glucose (34, 35). Hypocapnia has also been associated with neurotoxicity (12), by inducing the release of cytotoxic excitatory amino-acid (36), increasing dopamine levels in the basal ganglia (37) and by promoting the inappropriate incorporation of choline into the phospholipids of cell membranes (38).

## Controlled Hyperventilation in TBI Patients

Hyperventilation has been reported to effectively control ICHT in TBI patients (39, 40); in Table 2, a summary of most relevant studies reporting data on hypocapnia, ICP and outcome in this patients' population has been provided.

**Table 2:** List of most relevant clinical studies dealing with controlled hyperventilation in traumatic brain injury (TBI) patients.

**References**	**Aim of the study**	**Study design** **Study patients**	**Study population**	**Main results**	**Safety issues**
Cold	Association between hyperventilation and decreases of CBF below the ischemic threshold	Retrospective Single center 27	Comatose patients with TBI	Hyperventilation increased the number of areas with severe oligoemia Oligoemia was correlated to a poor outcome	Unsafe
Muizelaar et al. (41)	Effects of normo- and hyperventilation on the outcome	Prospective randomized interventional Single study 113	Patients >3 years old severe TBI	Hyperventilation was associated with poor outcome in 3 and 6 months	Unsafe
Carmona -Suazo et al. (42)	Effect of mild to moderate hyperventilation on cerebral oxygenation	Prospective observational Single center 90	Severe non-penetrating TBI	Increased hyperventilation caused a significant reduction in PbrO_2_	Probably unsafe
Coles et al. (2002) (43)	Effect of hyperventilation on CBF	Prospective interventional Single center 47	Non-penetrating TBI	Reduction of ICP and CBF Increase in HypoBV Normal global oxygenation parameters	NR
Diringer et al. (44)	Association between hyperventilation and CBF reduction and energy failure	Prospective interventional Single center 13	Adults severe TBI	Reduction on CBF No energy failure	Safe
Imberti et al. (45)	Effects of moderate hyperventilation on ICP, jugular venous oxygen saturation and PbtO_2_	Prospective interventional Single center 36	Patients>15 years severe non-penetrating TBI	Reductions of cerebral oxygenation (low PbtO_2_)	Unsafe
Marion et al. (46)	Potential adverse effects of brief periods of hyperventilation	Prospective interventional Single center 20	Severe TBI patients with surgical intracranial mass lesions	Increase in of cerebral glutamate, lactate, and lactate/pyruvate ratio in areas next to injured brain Reduction of CBF in some patients	Probably unsafe
Soustiel et al. (31)	Effects of moderate hyperventilation and mannitol on CBF and cerebral metabolic rates of oxygen, glucose and lactate	Prospective Single center 36	Adult severe TBI and ICP monitoring	Reduction of CBF and CMRO_2_ after hyperventilation Increase in anaerobic hyperglycolysis and lactate production	Unsafe
Mauritz et al. (47)	ICU management of TBI in Austria	Retrospective multicentric 145	Severe TBI	Aggressive hyperventilation were associated with poor ICU and 90-day outcomes Moderate hyperventilation was associated with better outcomes	Probably safe
Dumont et al. (48)	Inadequate ventilation and mortality in TBI	Retrospective Single center 77	Severe adult TBI patients	Hyper and hypoventilation were associated with increased in-hospital mortality	Unsafe
Rangel-Castilla et al. (49)	Effects of hyperventilation on cerebral hemodynamic	Prospective interventional Single center 186	Severe TBI patients	Reduction ICP, mean arterial pressure, jugular venous oxygen saturation, brain tissue oxygenation, and flow velocity	NR
Brandi et al.	Cerebral effects of moderate short-term hyperventilation	Prospective interventional Single center 11	Non-penetrating severe TBI adult patients Monitoring with ICP, PbtO_2_, and cMD	Decreased ICP Reduced PbtO_2_ but within normal ranges Cerebral glucose, lactate, and pyruvate unchanged	Safe
Tanaka et al. (50)	Association of ICP control management with neurological outcome	Retrospective Observational multicentric 195	Adult mild TBI patients	Hyperventilation was associated with poor outcome in 3 months	Unsafe
Svedung Wettervik et al. (51)	Cerebral effects of moderate short-term hyperventilation Outcome effects of moderate short-term hyperventilation	Retrospective observational Single center 120	Adult severe TBI patients Monitored with ICP and cMD	No effects on cerebral metabolism Hyperventilation was associated with better cerebral autoregulation indices	Safe
Zeiler et al.	Association between TIL for ICHT and cerebrovascular reactivity	Prospective Multicentric 249	Monitoring with ICP	Hyperventilation was associated with a modest improvement in cerebral autoregulation indices	NR

*CBF, cerebral blood flow; TBI, traumatic brain injury; PbrO_2_, regional brain oxygen pressure; ICP, intracranial pressure; HypoBV, hypoperfusion brain volume; PbtO_2_, brain tissue oxygenation; CMRO, cerebral metabolic rates of oxygen; cMD, continuous microdialysis; TIL, therapeutic intensity level; ICHT, intracranial hypertension; NR, not reported*.

Obrist et al. showed that hyperventilation could rapidly reduce ICP in half of TBI patients, although this was associated to a reduction in CBF in almost all of them (4). The relationship between PaCO_2_ and ICP is not linear and the most important effects are observed between PaCO_2_ values of 30 and 50 mmHg (52). Moreover, prolonged hyperventilation (53) will be associated with a progressive reduction of its vasoconstrictive effects, because of the perivascular normalization of pH due to local buffering. As reduced CBF (i.e., oligemia) is frequently observed in the early phase after TBI (54), prolonged hyperventilation should not be initiated in these patients without CBF monitoring. Cerebral blood flow can be measured directly, using Xenon computed tomography (CT) scan, CT perfusion (CTP) scan, or positron emission tomography (PET) scan, but these techniques involve injection of radioactive tracers or contrast media and require patients' transportation, which is not always feasible in severe TBI cases with ICHT (55). Indirect CBF velocities assessment using transcranial Doppler (TCD) ultrasonography does not directly correspond to absolute CBF values (56), although elevated pulsatility index (PI >1.2), low diastolic velocities in the middle cerebral artery (<20 cm/s) and estimated ICP using validated formulas might be helpful to identify TBI patients at risk of hypoperfusion. Different studies have shown a reduction in CBF levels during hyperventilation (5, 57–59); also, the most relevant reduction in CBF was observed in the peri-contusional areas, which are more vulnerable to secondary injuries (59).

If the reduction in CBF is quite consistent, controlled hyperventilation (i.e., mean PaCO_2_ from 37 to 30 mmHg) could improve indices of cerebral autoregulation function in TBI patients with disturbed pressure-reactivity at baseline, whereas those with intact pressure-reactivity at baseline would have no effect of such intervention (60). Another large cohort study also showed that mild hyperventilation was associated with lower pressure reactivity index (i.e., better autoregulatory function), in particular on day 2 after injury (61). One hypothesis is that hypocapnia and related vasoconstriction could reestablish endothelial reactivity in cerebral vessels, which were previously dilated in order to compensate for reduced cerebral oxygen delivery in the presence of ICHT.

Nevertheless, if a reduction in CBF is observed during hyperventilation, it remains unclear whether this phenomenon is associated with signs of cellular hypoxic injury and anaerobic metabolism. In severe TBI patients, Diringer et al. (62) observed that short and moderate hyperventilation significantly decreased CBF but did not impair global cerebral metabolism and oxygen extraction. As such, the use of neuromonitoring, in particular of cerebral oxygenation and/or metabolism, could provide important findings about the brain tolerance to controlled hyperventilation. Forced hyperventilation has been associated with reduced cerebral oxygenation, which was measured by the jugular bulb oximetry (SjO_2_, i.e., the threshold for cerebral hypoxia being <55%), although these results were not consistent in all studies (63–65). However, SjO_2_ reflect hemispheric global oxygenation and tissue hypoxia may occur even within normal SjO_2_ values (66). Brain tissue oxygen tension (PbtO_2_) is a regional technique, is well-correlated with local CBF and can directly monitor the areas at higher-risk of secondary ischemia (67). The effects of hyperventilation on PbtO_2_ are variable, with some studies reported a significant reduction in brain oxygenation (68–71) while others showing no major changes (72, 73) and some reporting an increase in PbtO_2_, in particular due to the large reduction in ICP with previous cerebral vasodilation (i.e., hyperemia) (45, 74). Recent studies reported unchanged PbtO_2_ values in adult severe TBI patients undergoing moderate hyperventilation and with a median PbtO_2_ value at baseline within normal values (i.e., >30 mmHg) (39, 40).

Cerebral metabolic function can be assessed at bedside using the microdialysis technique, or performing PET and magnetic resonance imaging (MRI) spectroscopy studies. In one study, early hyperventilation (i.e., 24–36 h after injury) was associated with a significant increase in tissue lactate and lactate/pyruvate ratio, suggesting anaerobic metabolism and tissue hypoxia (46); these metabolic effects were less pronounced at a late phase (i.e., 3–4 days after TBI). However, two recent studies showed no effect of moderate hyperventilation on cerebral metabolites in adult TBI patients (39, 40). Using PET scan, one study (*n* = 9) showed that moderate and intense hyperventilation resulted in reduced CBF and increased oxygen extraction, but a constant oxygen metabolism (i.e., no energy failure) (44). In two larger studies (43, 75), hyperventilation (i.e., PaCO_2_ <30 mmHg) increased the volume of hypoperfused cerebral areas within the injured brain, which, in the absence of increased oxygen extraction, would result in tissue hypoxia.

With all these potential side effects, which is the effect of controlled hyperventilation on the outcome of TBI patients? In one large retrospective study (*n* = 251), Gordon et al. (76) reported a lower mortality in TBI patients undergoing hyperventilation (i.e., PaCO_2_ between 25 and 30 mmHg for 6 to 41 days); however, more severe neurological sequelae were observed among survivors in the hyperventilation group when compared to the other. Only one prospective randomized clinical trial has investigated the effects of hyperventilation in this setting; Muizelaar et al. (41) compared the 3- and 6-month neurological outcome of patients who were kept at a median PaCO_2_ of 25 mm Hg to those kept at a median of 35 mmHg for 5 days: forced hyperventilation was associated with a higher proportion of patients with poor outcome. Interestingly, among patients being treated with controlled hyperventilation and tromethamine (THAM, i.e., a buffer that prevents pH changes within the extracellular cerebral fluid and excessive vasoconstriction), there was a higher proportion of patients with long-term favorable neurological outcome when compared to the others.

## Discussion: A Practical Approach

**Figure 1:**
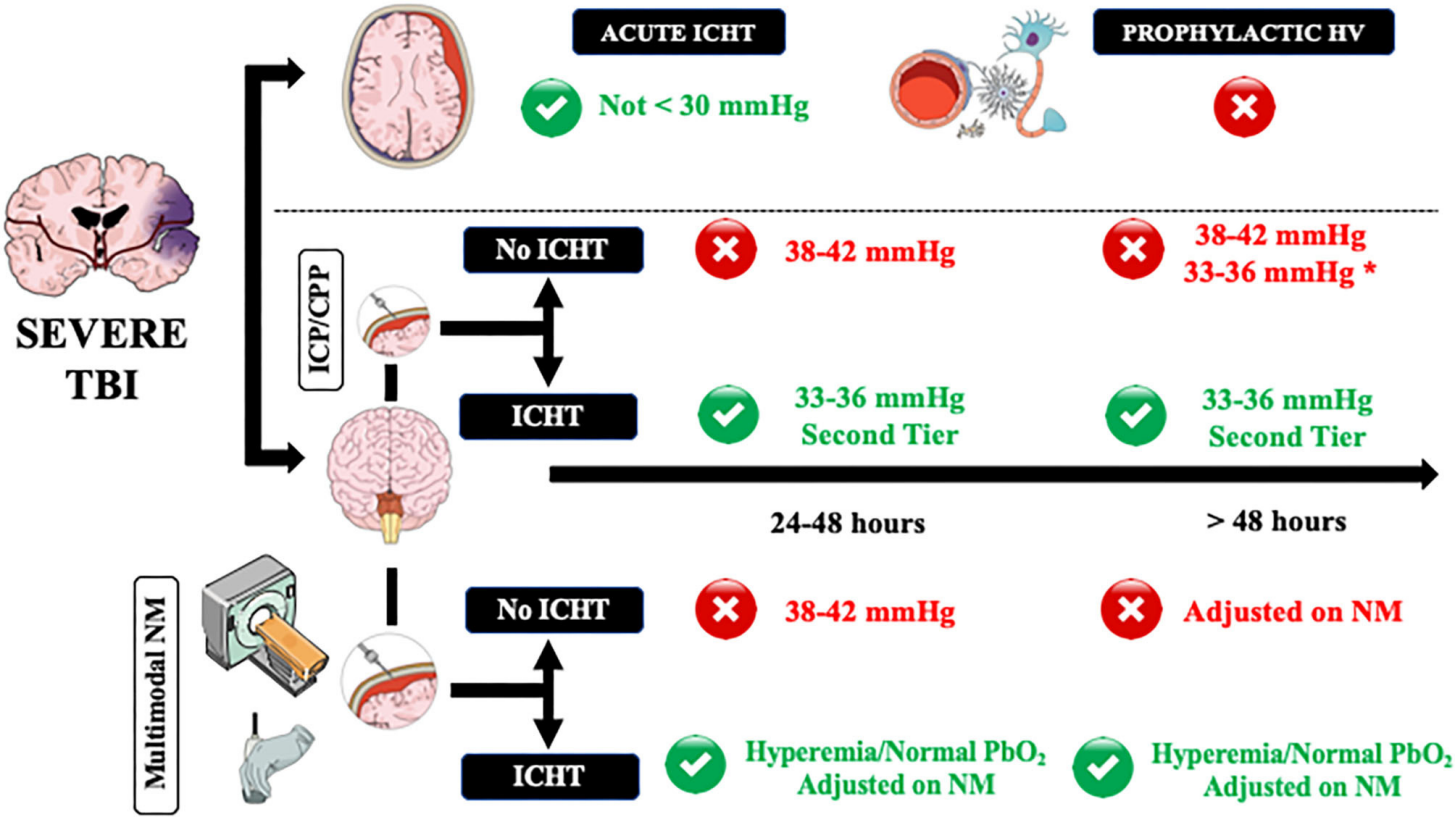
A practical approach on how to manage controlled hyperventilation and hypocapnia in traumatic brain injury (TBI) patients. Acute intracranial hypertension (ICHT) = life-threatening elevation in ICP, in particular when signs of herniation (i.e., anisocoria, apnea, hypertension, bradycardia) are present. ICHT, intracranial pressure close to the critical threshold for therapy without signs of herniation; HV, controlled hyperventilation and hypocapnia. Green and red circles refers to the potential use (green) or contraindication (red) to the use of HV. NM, neuromonitoring. *In case of diffuse brain injury but with high potential risk of tissue hypoxia. **Adjusted on neuromonitoring.

The initial PaCO_2_ targets in TBI patients with normal ICP values undergoing mechanical ventilation should be within normal values (i.e., 38–42 mmHg—Figure 1); although Brain Trauma Foundation guidelines could not identify an optimal threshold for PaCO_2_ values in the initial phase of TBI management [(66), international consensus recommended for these “physiological” values as oligemia is frequent in the first 24–48 h after injury and could be aggravated by hypocapnia (49, 50). Prophylactic (i.e., in the absence of ICHT) and prolonged hyperventilation is not recommended and should not be used, (61) as it would provide no benefits and could result in tissue hypoxia and cerebral metabolic disturbances. In order to detect cerebral oligemia in these patients, an initial CTP scan could be helpful to identify very low CBF values, which would result in secondary ischemia in case PaCO_2_ would decrease below physiological values. In the absence of CTP, cerebral ultrasound, using a combination of PI, estimated ICP and diastolic CBF velocity, could identify patients at risk of cerebral hypoperfusion. A close attention on gas analyses monitoring, requiring repeated sampling and end-tidal CO_2_ (etCO_2_) monitoring, is necessary in this phase, as hyperventilation in the absence of elevated ICP is frequently observed in adult TBI patients (61). Importantly, etCO_2_ might have some limitations in case of concomitant severe chest trauma and hemodynamic instability (i.e., low cardiac output) (77). Whether targeting normal PaCO_2_ or pH values would be the most appropriate approach remains unknown in these patients. However, as cerebral perivascular pH could be influenced by other factors than PaCO_2_ and systemic pH (i.e., local metabolism, K^+^, isolated cerebral hypoxia) and therefore be less accurately predicted, using PaCO_2_ levels and quantifying changes in cerebral hemodynamics and physiology to PaCO_2_ changes at bedside is more feasible for physicians. If ICP remains within acceptable values after 48 h from the injury (i.e., <15 mmHg), lower PaCO_2_ values (i.e., 33–36 mmHg) could result in improved cerebral autoregulation (66), however it is hard to recommend this approach routinely in all severe TBI patients. TBI patients at the highest risk of impaired cerebral autoregulation are those with diffuse brain injury (51); as such, if TCD assessment shows normal CBF velocities in these patients, lower PaCO_2_ values (i.e., 33–36 mmHg) could be tolerated, although a more comprehensive neuromonitoring would be the only effective solution to detect the potential occurrence of tissue hypoxia.

As hyperventilation combined with hypocapnia is the most rapidly available method to reduce ICP, moderate and brief hyperventilation should be used to treat life-threatening elevation in ICP, in particular when signs of herniation (i.e., anisocoria, apnea, hypertension, bradycardia) are present (61) and could be used as a bridge toward additional interventions (i.e., repeated CT-scan; osmotic therapy; surgery). In this setting, hyperventilation should be of short duration and, if possible, should never decrease below PaCO_2_ values of 30 mmHg because: (a) the most important effects of PaCO_2_ on ICP are observed between 30 and 50 mmHg and (b) most of the relevant cerebral side effects were reported for forced hyperventilation. If possible, the use of hyperventilation should be minimized for these patients during the first 24 h after injury, when CBF often is the most reduced (61), unless CBF could be measured.

In patients with ICP values remaining close to the critical threshold for therapy (i.e., 20–22 mmHg), international guidelines recommended the use of controlled hyperventilation only as “tiers 2” therapy, after the failure of increased sedation and osmotics infusion (78); indeed, controlled hyperventilation produced similar effects on ICP but more metabolic disturbances of cerebral metabolism than mannitol in TBI patients (61). In these patients, it is recommended to set PaCO_2_ around 33–36 mmHg and avoid values <30 mmHg (49). In the absence of other neuromonitoring than ICP and cerebral perfusion pressure (CPP), CTP scan and TCD are helpful to suggest normal or high (i.e., hyperemia) CBF values, which would logically respond to hyperventilation. However, in case of oligemia, physicians could decide to avoid hypocapnia and move to “tiers 3” therapy, such as barbiturates, hypothermia, or decompressive craniectomy, to treat ICHT as hypocapnia would result in additional cerebral hypoperfusion.

In our opinion, invasive neuromonitoring should be also considered in severe TBI patients and ICHT to optimize overall management and, in particular, to assess the effects of low PaCO_2_ values on cerebral oxygenation and metabolism. As such, PaCO_2_ values and hyperventilation could be adjusted to the brain tolerance and therapeutic targets individualized on the patients' need. In case of the need for low PaCO_2_ values and concomitant lung injury, other interventions aiming at reducing CO_2_ production, such as increasing sedation or hypothermia, could be considered to induce hypocapnia and avoid lung stress and VILI. However, this strategy should be further evaluated in clinical studies.

If controlled PaCO_2_ values are mandatory in severe TBI patients because of the significant effects on brain hemodynamics and compliance, high doses of sedatives, often in association with neuromuscular blocking agents (NMBAs), are required to adjust ventilatory parameters to obtain desired PaCO_2_ targets. As such, in the absence of aggressive therapies for ICHT, it remains unknown when it would be safe to discontinue sedatives and eventually tolerate spontaneous hyperventilation in these patients. If this occurs after several (i.e., >7–10) days from injury, the risk of oligemia would be probably limited. Also, spontaneous hyperventilation after acute brain injury is often triggered by local acidosis (i.e., low pH surrounding the respiratory center located in the brainstem), which result in vascular dilation and would probably compensate from vasoconstriction induced by hyperventilation. In these cases, non-invasive (i.e., TCD) and invasive (i.e., PbtO_2_) monitoring would again be helpful to individualize therapeutic decisions.

In conclusions, controlled hyperventilation is effective in reducing ICP but it also reduces CBF and might have both cerebral and systemic serious side effects. As such, normal PaCO_2_ values should be maintained in the early phase after TBI if ICP remains within acceptable values. Controlled hyperventilation (i.e., never below PaCO_2_ of 30 mmHg) should be used as a temporary life-saving intervention in case of severe intracranial hypertension; PaCO_2_ levels should be also adjusted and individualized in each patient using CTP and cerebral ultrasound of, whenever possible, advanced multimodal neuromonitoring.

## Author Contributions

EG, LP, JC, and FT contribute equally to the elaboration of this manuscript. All authors contributed to the article and approved the submitted version.

## Conflict of Interest

The authors declare that the research was conducted in the absence of any commercial or financial relationships that could be construed as a potential conflict of interest.
